# 

**Published:** 1882-07

**Authors:** 


### JULY, 1882.—CONTENTS.

PAGE

ORIGINAL COMMUNICATIONS—Tuberculosis in Men and Animals. Thomas E. Satterthwaite, M.D. . . . . . . . . . . 203

The Physiology of Digestion in Ruminants, with Practical Remarks. Henry C. Slee. . . . . . . . 235

Practical Suggestions to Young Veterinarians. Ezra M. Hunt, M.D. . . . . . . . . 241

Purulent Arthritis. William Henry Porter, M.D., D.V.S. . . . . . . . . 245

EDITORIAL—Protecting Animals by Vaccination—The Cause of Bovine Tuberculosis—The Effect of Various Medicines upon the Milk Secretion—How much does the Horse's Hoof Expand?—A New Cause for Bloody Urine in Horses—Digestion in Ruminant Animals—The Identity of Bovine and Human Tuberculosis—Monstrosities in General: A Double-faced Horse—Paralysis in Cows and Hæmoglobinuria in Horses from the Same Cause . . . . . . . . . 249–254

CASE DEPARTMENT—Gangrena Oris in a Python: A New Form of Bothrocephalus—Intussusception of the Ilium in a Two-year-old Stallion—Suppurative Nephritis in a Young Dromedary—Acute Lead Poisoning in a Cow—Acute Gastro-Entoritis from an Overdose of Sodium Chloride—Tuberculosis in a Cow—A Case of Colic followed by Enteritis Ulceration of Stomach and Possible Perforation—Impaction of the Colon in a Horse—Flatulent Colic in a Horse—Lacerated Wound of the Neck in a Horse—A Novel Way of Feeding Pups. . . . . . . . . . 255–261

REVIEWS—The Simple Ailments of Horses: Their Nature and Treatment—Books and Pamphlets Received . . . . . . . . . . 262–263

PROGRESS OF VETERINARY SCIENCE—Typhoid Fever in a Horse—An Ossifying Spinal Pachymeningitis in a Dog—Hair-balls in a Ten days old Calf—Dilated and Calcified Arteries in a Parrot Sixty-four Years Old—Death from Asphyxia in a Swan—Gall-stone in a Horse—Health of Dr. C. D. House—Syringe for Veterinary Use—Kidney Disease in the Cow—Bile Pigments in the Urine of the Dog—Experiments with Huch's Kraft-futter or Blood-meal on Horses—Epidemic Nose Bleeding and Pernicious Anæmia in Dogs—Remedy for Lice—Goitre in the Lower Animals—Diphtheria in Calves Communicated to Pigs—Effect of Arsenic on Cats—Human and Bovine Tuberculosis—Resuscitation of Animals after Exposure to Extreme Cold. . . . . . . . . . 264–267

NEWS AND MISCELLANY—Death of Professor David—Royal College of Veterinary Surgeons—Edinburgh Veterinary College—Royal Agricultural Society—Cæsarian Section in a Cow—Decrease of Sheep in England—Consumption of Horse Flesh—Lecture on Horses—Horse-Cleaning Machine—Animals that Drink Rum—Ways in Which Milk May be Affected—Dogs Appreciate Medical Treatment—Cemetery for Cats—Bird's-Nest in a Horse's Tail—To Keep Flies from Horses—The (Veterinary) Anatomist to his Dulcinea—Turkish Baths for Horses—Cruelty to a Horse Punished—Duck-Rearing in China—Toothsome Python Eggs—The British National Veterinary Congress—Influence of Race upon the Action of Poison—The Salmon Disease—A Hermaphrodite Hog—Live Stock in the United States—Dr. H. J. Detmers—Pet Animals as Carriers of Contagion—The Early Development of Fast Trotters—Stamping Out Disease—A Monstrosity—A Freak of Nature . . . . . 268–279

